# Association of Interleukin-32 and Interleukin-34 with Cardiovascular Disease and Short-Term Mortality in COVID-19

**DOI:** 10.3390/jcm12030975

**Published:** 2023-01-27

**Authors:** Christoph C. Kaufmann, Amro Ahmed, Marie Muthspiel, Isabella Rostocki, Edita Pogran, David Zweiker, Achim Leo Burger, Bernhard Jäger, Gabriele Aicher, Alexander O. Spiel, Florian Vafai-Tabrizi, Michael Gschwantler, Peter Fasching, Johann Wojta, Kurt Huber

**Affiliations:** 1 3rd Medical Department with Cardiology and Intensive Care Medicine, Klinik Ottakring (Wilhelminenhospital), 1160 Vienna, Austria; 2 Department of Endocrinology and Rheumatology, Klinik Ottakring (Wilhelminenhospital), 1160 Vienna, Austria; 3 Division of Cardiology, Medical University of Graz, 8036 Graz, Austria; 4 Department of Laboratory Medicine, Klinik Ottakring (Wilhelminenhospital), 1160 Vienna, Austria; 5 Department of Emergency Medicine, Klinik Ottakring (Wilhelminenhospital), 1160 Vienna, Austria; 6 2nd Medical Department with Pneumology and Karl-Landsteiner-Institute for Lung Research and Pulmonary Oncology, Klinik Ottakring (Wilhelminenhospital), 1160 Vienna, Austria; 7 Department of Gastroenterology and Hepatology, Klinik Ottakring (Wilhelminenhospital), 1160 Vienna, Austria; 8 Medical School, Sigmund Freud University, 1020 Vienna, Austria; 9 Department of Internal Medicine 2, Division of Cardiology, Medical University of Vienna, 1090 Vienna, Austria; 10 Core Facilities, Medical University of Vienna, 1090 Vienna, Austria; 11 Ludwig Boltzmann Institute for CV Research, 1090 Vienna, Austria

**Keywords:** interleukin-32, interleukin-34, CV disease, COVID-19, short-term mortality

## Abstract

Background: Excess cardiovascular (CV) morbidity and mortality has been observed in patients with COVID-19. Both interleukin-32 (IL-32) and interleukin-34 (IL-34) have been hypothesized to contribute to CV involvement in COVID-19. Methods: This prospective, observational study of patients with laboratory-confirmed COVID-19 infection was conducted from 6 June to 22 December 2020 in a tertiary care hospital in Vienna, Austria. IL-32 and IL-34 levels on admission were collected and tested for their association with CV disease and short-term mortality in patients with COVID-19. CV disease was defined by the presence of coronary artery disease, heart failure, stroke or atrial fibrillation and patients were stratified by CV disease burden. Results: A total of 245 eligible patients with COVID-19 were included, of whom 37 (15.1%) reached the primary endpoint of 28-day mortality. Of the total sample, 161 had no CV disease (65.7%), 69 had one or two CV diseases (28.2%) and 15 patients had ≥three CV diseases (6.1%). Median levels of IL-32 and IL-34 at admission were comparable across the three groups of CV disease burden. IL-32 and IL-34 failed to predict mortality upon both univariable and multivariable Cox regression analysis. The two CV disease groups, however, had a significantly higher risk of mortality within 28 days (one or two CV diseases: crude HR 4.085 (95% CI, 1.913–8.725), *p* < 0.001 and ≥three CV diseases: crude HR 13.173 (95% CI, 5.425–31.985), *p* < 0.001). This association persisted for those with ≥three CV diseases after adjustment for age, gender and CV risk factors (adjusted HR 3.942 (95% CI, 1.288–12.068), *p* = 0.016). Conclusion: In our study population of hospitalized patients with COVID-19, IL-32 and IL-34 did not show any associations with CV disease or 28-day mortality in the context of COVID-19. Patients with multiple CV diseases, however, had a significantly increased risk of short-term mortality.

## 1. Introduction

Severe acute respiratory syndrome coronavirus 2 (SARS-CoV-2) was first reported late December 2019, in Wuhan, China, and has since spread across the globe causing an international pandemic [1]. However, the original spread of SARS-CoV-2 may have started as early as September 2019 [2]. Coronavirus disease 2019 (COVID-19) imposes a unique challenge on the global economy, on everyday social life and on treating physicians with regard to individual patient care [3]. According to data from the World Health Organization (WHO), a total of 663 million COVID-19 cases have been reported since the beginning of the pandemic, with a case fatality rate of approximately 1%, which has resulted in 6.7 million lethal cases of COVID-19 [4]. Ongoing research demonstrated that COVID-19 is a multi-system disease affecting several organ systems of the human body; as such, cardiovascular (CV) involvement was described shortly after the outbreak [5]. Since then, a growing body of evidence has further underlined the necessity of a special focus on the CV system during early management of patients with COVID-19, considering the high rates of adverse CV events [6,7]. Patients with COVID-19 and a history of CV disease in particular, as well as those with increased levels of CV biomarkers—most notably high-sensitivity cardiac troponin I (hs-cTnI) and N-terminal pro-B-type natriuretic peptide (NT-proBNP)—face an increased risk of morbidity and mortality. In addition to cardiovascular disease, thromboembolic complications, such as deep-vein thrombosis or pulmonary embolism, have also been encountered in COVID-19 [8]. COVID-19 has been strongly established as a multiorgan disease in general, with the virus leading to complications such as pre-eclampsia, male infertility and brain damage, among many others [9,10,11]. While several theories have emerged offering potential explanations for the association of CV disease with COVID-19, the direct mechanism has not been elucidated yet [12,13,14].

The concept of a cytokine storm has been suggested as one of the major contributors to the pathophysiological cascade of severe COVID-19 [15]. Several studies showed a significant increase in inflammatory cytokines, such as IL-3, IL-6 and IL-10, among patients with a severe or lethal disease course of COVID-19 [16,17,18]. Targeting this pathway, the use of the IL-6 inhibitor, tocilizumab, resulted in a reduction in clinical endpoints in hospitalized patients with COVID-19. Tocilizumab was one of the first targeted treatment options for COVID-19 with data emerging early during the pandemic [19]. Recently, Law et al. hypothesized a potential role of IL-32, IL-34 and IL-37 for the manifestation of CV complications in COVID-19 [20]. The authors argued that COVID-19—severe cases in particular—promotes atherosclerosis through the development of a cytokine storm and subsequent activation of endothelial cells. This may in turn be associated with IL-32 and IL-34 as both biomarkers have been shown to contribute to the process of atherosclerosis, IL-32 mostly through the promotion of angiogenesis on endothelial cells and modification of lipid profiles, and IL-34 through its association with obesity, chronic inflammation and insulin resistance [21,22,23]. Moreover, a small study of 64 patients with COVID-19 showed that concentrations of IL-32 were lower in patients with COVID-19 compared to healthy controls. However, no significant difference was found according to the severity of COVID-19 infection [24].

Thus, the primary aim of our study was to evaluate an association between IL-32/IL-34 with CV disease in the context of COVID-19. We also assessed the prognostic impact of IL-32/IL-34 and CV disease on short-term mortality in our well-defined study population of hospitalized patients with COVID-19.

## 2. Materials and Methods

### 2.1. Study Design and Patient Population

**Figure 1:**
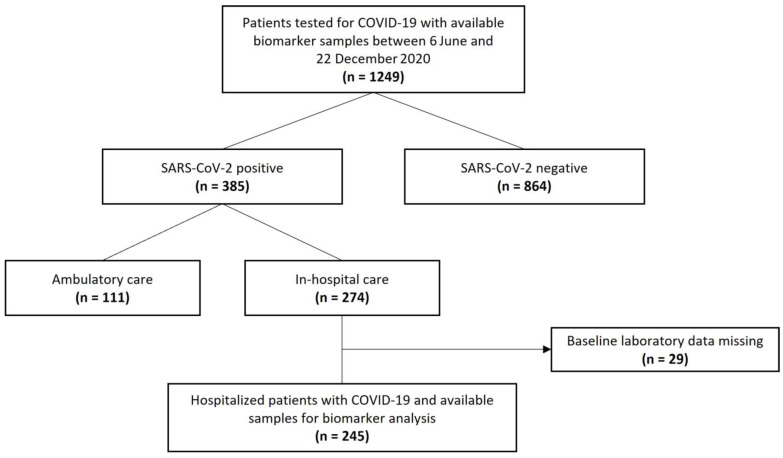
Study flow chart.

A total of 245 hospitalized patients with laboratory-confirmed SARS-CoV-2 infection and available blood samples for biomarker analysis were included in this prospective, observational, single-center study in different departments of Clinic Ottakring, a tertiary hospital in Vienna, Austria. All patients presented to the emergency department between 6 June and 22 December 2020 and were admitted for in-hospital care. The indication for hospitalization was made by the treating physician, who did not have information on levels of IL-32 or IL-34 as these biomarkers were measured retrospectively at a later time point. Only patients presenting to the emergency department with a decision for hospitalization and a positive SARS-CoV-2 test were available for inclusion in the study. Patients under 18 were excluded from study participation. Patients with missing baseline laboratory values or insufficient sample volumes were also excluded (Figure 1). The diagnosis of COVID-19 was made by direct detection of SARS-CoV-2 RNA upon polymerase chain reaction on nasal and/or pharyngeal swabs. This is in line with recommendations of the World Health Organization (WHO) [25]. The study was approved by the local ethics committee of the city of Vienna (EK 20-100-VK) and complies with the Declaration of Helsinki and the International Conference on Harmonization Guidelines for Good Clinical Practice.

Details on data collection have been published previously in two papers from a similar study population. In the first paper, we showed a significant association between mid-regional pro-atrial natriuretic peptide (MR-proANP) and short-term mortality in COVID-19. The second paper suggested a dual-biomarker approach, using both high-sensitivity troponin I and copeptin for outcome prediction in COVID-19 [26,27].

Demographic data, clinical features, laboratory results and medical history were obtained from patient records. CV disease was defined by a history of coronary artery disease (CAD), heart failure, stroke or atrial fibrillation. Patients were then stratified into three groups based on the burden of CV disease: no CV disease, one or two CV diseases and ≥three CV diseases. Chronic pulmonary disease was defined by a history of chronic obstructive pulmonary disease, bronchial asthma or obstructive sleep apnea. Comorbidities were defined at the discretion of the treating physician. In addition to the aforementioned variables, we also analyzed electrocardiograms (ECGs) upon admission. ST-segment deviation comprises either T-wave inversion, ST-segment depression or ST-segment elevation. The Horowitz index (pO2/FiO2) was calculated from blood gas analysis data when available (n = 213). Follow-up data was collected through the electronic patient record system of our institution until 19 January 2021. The primary endpoint of our study was short-term all-cause mortality within 28 days.

### 2.2. Biomarker Analysis

Serum blood samples were drawn by trained nurses or doctors upon presentation of the patient to the emergency department. After centrifugation, serum blood samples for biomarker analysis were divided into 0.5 mL aliquots and immediately stored at −80 °C until measurement. IL-32 and IL-34 were measured using commercially available ELISA kits (Human IL-32 alpha ELISA Kit, ThermoFisher Scientific, article number EH274RBX5 and Human IL-34 ELISA Kit, ThermoFisher Scientific, article number EH275RBX5) according to the manufacturer’s protocol.

IL-32 and IL-34 were measured as part of the research protocol months after admission of the patients and the treating physicians were therefore blinded to the results. The other laboratory markers (e.g., hs-cTnI or NT-proBNP) were measured during clinical routine and were, hence, available to the treating physician. Routine assessment of laboratory values was performed at the certified central laboratory of the Clinic Ottakring and included white blood cells, neutrophil granulocytes, lymphocytes, C-reactive protein (CRP), platelets, hemoglobin, creatinine, sodium, potassium, LDH, Nt-proBNP and hs-cTnI, respectively. The neutrophil-to-lymphocyte ratio (NLR) was calculated by dividing the absolute neutrophil count by the absolute lymphocyte count.

### 2.3. Statistical Analysis

The primary outcome of our study was the association between cardiovascular disease and IL-32/IL-34 in COVID-19. Baseline characteristics and diagnostic findings were compared across patients with CV disease (either one/two CV diseases or ≥three CV diseases) and those without CV disease. Continuous data are reported as median and interquartile range (IQR), and categorical data are expressed as frequency and percentage. The Kruskal–Wallis test and Pearson’s chi-squared test were used to compare continuous and categorical data between patients with CV disease and those without CV disease. Bonferroni correction was used to account for multiple testing.

Associations between cardiovascular biomarkers (NT-proBNP and hs-cTnI) and IL-32/IL-34 were evaluated using Spearman correlation analysis. Kaplan–Meier survival analysis was used to compare 28-day mortality across different tertiles of IL-32 and IL-34. We also checked for differences in survival in individuals with CV disease and those without as well as for the individual determinants of CV disease (CAD, heart failure, stroke and atrial fibrillation). The log rank test was utilized to compare the difference of curves. Additionally, both unadjusted and adjusted regression models were used to analyze the association between IL-32, IL-34 and CV disease with 28-day mortality. First, univariable Cox regression analysis was performed, followed by multivariable Cox regression analysis with adjustments for age and gender (Model 1) as well as for age, gender and comorbidities (Model 2).

All statistical analyses were performed using SPSS 27.0 (SPSS Inc., Chicago, IL, USA). Graphics were generated using GraphPad Prism 9.0 (GraphPad Software, Inc., San Diego, CA, USA). A two-sided *p*-value < 0.05 was required for statistical significance.

## 3. Results

### 3.1. Clinical Characteristics and Findings of Diagnostic Work-Up

The final study population encompassed 245 hospitalized patients with COVID-19, of whom 37 met the primary endpoint of 28-day mortality (15.1%). The median age was 67 years and 53.1% of the patients were male. The proportion of patients with one or two CV diseases was 28.2% (n = 69) and the proportion with ≥ 3 CV diseases was 6.1% (n = 15) (Table 1 and Table 2).

Patients with CV diseases were significantly older and had a higher comorbidity burden (arterial hypertension, chronic kidney disease and history of malignancy) compared to those without CV disease. Within the subgroup of patients with CV disease, 48.8% had CAD (n = 41), 31.0% heart failure (n = 26), 20.2% a history of stroke (n = 17) and 61.9% atrial fibrillation (n = 52). While pulmonary hypervolemia upon chest X-ray at admission was significantly more often found in patients with CV disease, we did not observe any differences with regard to pulmonary infiltrates. Routine laboratory markers also differed significantly between both groups with higher levels of neutrophil granulocytes, NLR, as well as creatinine and lower levels of hemoglobin and lymphocytes among the groups of patients with CV diseases. The 28-day mortality rate was significantly higher in both CV disease groups. PO2/FiO2 levels were similar between the three study groups.

Detailed baseline characteristics and diagnostic findings of the study population can be found in Table 1 and Table 2.

### 3.2. Correlation and Association of IL-32/IL-34 with CV Disease and Biomarkers of CV Disease

**Figure 2:**
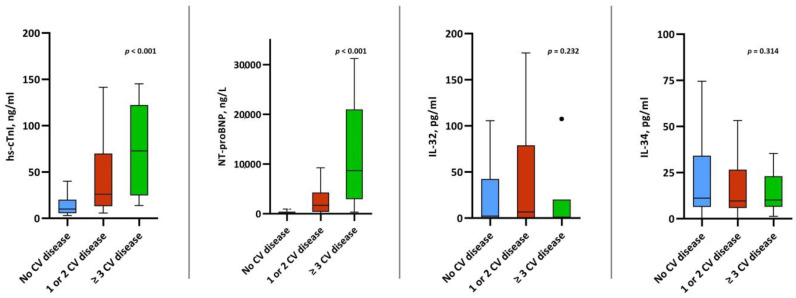
Box plots of IL-32, IL-34, hs-cTnI and NT-proBNP levels stratified by CV disease.

Plasma levels of both IL-32 and IL-34 were comparable among patients with CV disease compared to those without CV disease (IL-32: 2.2 pg/mL (IQR, 0.0–41.1) vs. 6.7 pg/mL, (IQR, 0.0–73.6) vs. 1.0 pg/mL (0.0–20.10, *p* = 0.232; and IL-34: 11.1 pg/mL (IQR, 6.4–33.9) vs. 9.6 pg/mL (IQR, 6.1–25.7) vs. 10.1 pg/mL (IQR, 6.5–23.0), *p* = 0.314). Contrarily, we observed significantly higher levels of hs-cTnI and NT-proBNP among those with a history of CV disease (Figure 2). While there was a significant correlation between IL-32 and IL-34 (r = 0.332), neither of the interleukins showed any correlation with hs-cTnI or NT-proBNP (Table 3). We also did not observe a correlation between levels of IL-32/IL-34 and pO2/FiO2 (IL-32: r = −0.067, *p* = 0.329 and IL-34: r = −0.024, *p* = 0.727).

### 3.3. Association of IL-32/IL-34 and CV Disease with the Primary Endpoint

**Figure 3:**
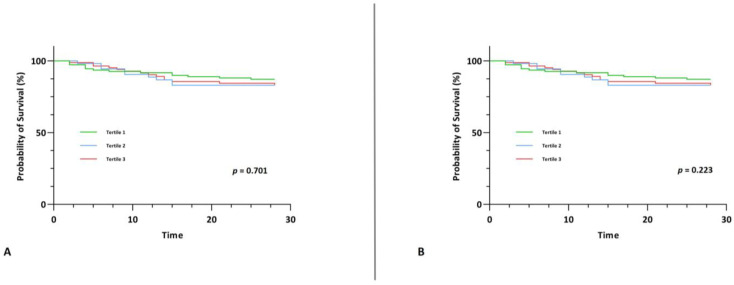
Kaplan-Meier survival curve for the primary endpoint of 28-day mortality stratified by tertiles of IL-32 (**A**) and by tertiles of IL-34 (**B**).

IL-32 and IL-34 did not show any associations with short-term mortality upon univariable and multivariable Cox regression analysis (Table 4). We also did not observe any difference between survival among tertiles of IL-32 and IL-34 upon Kaplan-Meier survival analysis (χ^2^(2) = 0.712, *p* = 0.701 and χ^2^(2) = 2.999, *p* = 0.223) (Figure 3).

**Figure 4:**
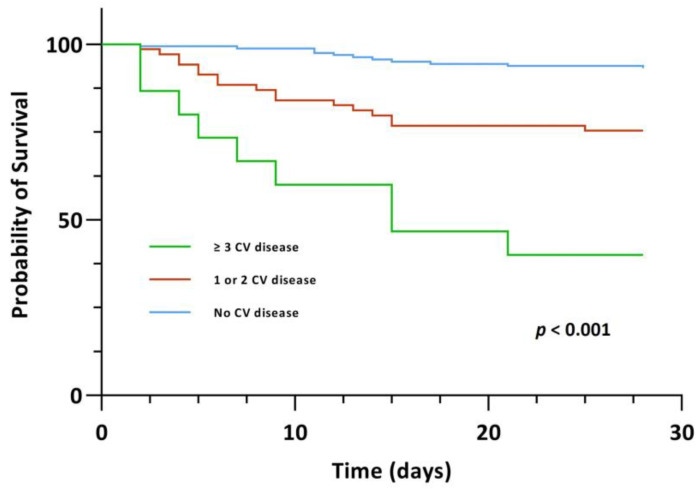
Kaplan-Meier survival curve for the primary endpoint of 28-day mortality stratified by CV disease.

**Figure 5:**
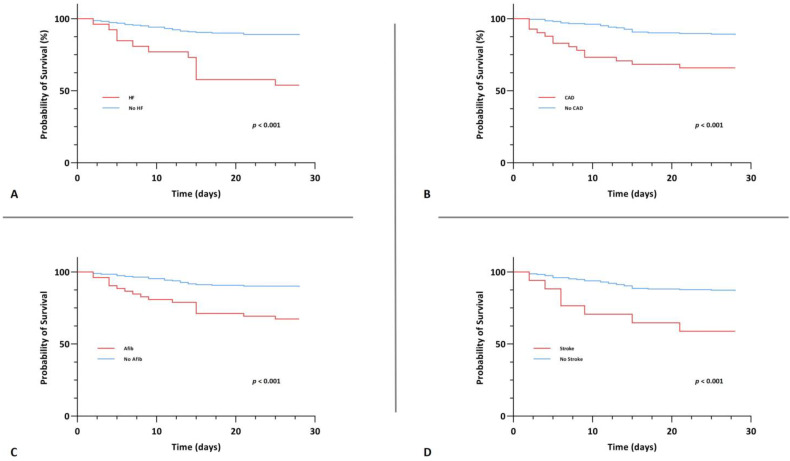
Kaplan-Meier survival curve for the primary endpoint of 28-day mortality stratified by coronary artery disease (**A**), heart failure (**B**), atrial fibrillation (**C**) and stroke (**D**).

A significant association between CV disease, both those with one/two CV diseases and those with ≥three CV diseases, and 28-day mortality, however, was evident upon univariable Cox regression analysis (crude HR 4.085 (95% CI, 1.913–8.725), *p* < 0.001 and crude HR 13.173 (95% CI, 5.425–31.985), *p* < 0.001). In the group of patients with ≥three CV diseases, this association persisted upon multivariable Cox regression analysis after adjusting for age and gender (Model 1: adjusted HR 4.013 (95% CI, 1.443–11.157), *p* = 0.008) as well as after adjusting for age, gender and comorbidities (Model 2: adjusted HR 3.959 (95% CI, 1.304–12.016), *p* = 0.015). Kaplan-Meier survival analysis also showed that the CV disease groups exhibited significantly lower 28-day survival (χ^2^(2) = 46.590, *p* < 0.001) (Figure 4). The survival distributions for CAD, heart failure, stroke and atrial fibrillation on an individual level, also differed significantly (CAD: χ^2^(2) = 16.965, *p* < 0.001; heart failure: χ^2^(2) = 24.267, *p* < 0.001; stroke: χ^2^(2) = 11.640, *p* < 0.001 and atrial fibrillation: χ^2^(2) = 17.215, *p* < 0.001) (Figure 5).

## 4. Discussion

This prospective, observational single-center study (1) examined levels of IL-32 and IL-34 at hospital admission in a sizeable cohort of patients with COVID-19 for the first time, (2) found no association between IL-32/IL-34 and CV disease in the context of COVID-19 and (3) disproved a correlation between IL-32/IL-34 and outcome, while a higher CV disease burden predicted short-term mortality in hospitalized patients with COVID-19.

In the early phase of the pandemic, reports surfaced showing excess myocardial injury and CV morbidity in patients with COVID-19 [28]. Since then, several potential theories emerged trying to elucidate the pathophysiological mechanisms linking CV disease and COVID-19 [29,30]. One of these alludes to the theory that COVID-19 contributes to the process of atherosclerosis through initiation of vascular dysfunction by a cytokine storm and up-regulation of the Angiotensin-converting-enzyme-2 pathway, which subsequently leads to the activation of endothelial vascular cells. Additionally, increased plaque instability has been observed in the context of COVID-19, which further adds to the potential role of atherosclerosis in these patients [31]. IL-32 and IL-34 have recently been identified to play a role in specific patient populations with cardiovascular disease. As such, recent research data has identified IL-32 as a potential new biomarker of pulmonary arterial hypertension and an association with CAD has also been shown [22,32]. Similarly, IL-34 has been established as a prognostic biomarker in both CAD and heart failure, alike [33,34]. Law et al. recently hypothesized that IL-32, IL-34 and IL-37 may contribute to CV manifestations in COVID-19 by promoting a variety of clinical pathways. IL-32 has been shown to promote angiogenesis on endothelial cells and leads to a modification of lipid profiles, linking it directly to atherosclerosis [35,36]. IL-34 has been linked to obesity, chronic inflammation and insulin resistance, which in turn is associated with atherosclerosis [37]. Additionally, IL-34 induction has been observed in patients with influenza infection, which may also be a potential pathophysiological link to COVID-19 [38]. As this review clearly showed plausible mechanisms, it needed confirmation by clinical data [20].

Our study results, ultimately, do not support the hypothesis of Law et al. as levels of both IL-32 and IL-34 were comparable among patients with and without CV disease, even when taking into account the burden of CV diseases. We also did not find any correlations between the two interleukins and hs-cTnI/NT-proBNP, both surrogate markers of CV disease. To the authors’ knowledge, this is the first study clinically investigating IL-32 and IL-34 levels and associations with clinical outcome in patients with COVID-19. Despite a plethora of research data, the definite pathophysiological mechanism of CV involvement in COVID-19 remains elusive more than two years into the pandemic. This question should remain an important topic of research, as excess CV morbidity is a driver of mortality in patients with COVID-19, as again shown in this analysis.

Several studies have described the phenomena of a profound cytokine storm at the heart of the pathophysiology of severe and lethal COVID-19 [15]. Upon entry of respiratory epithelial cells, SARS-CoV-2 causes an acute immune response with inflammatory cytokine production and interferon response. As such, upregulation of IL-6 and TNF-α are core components of the cytokine storm in COVID-19 [39]. Various levels of cytokines release patterns have been shown in COVID-19 with excess cytokine release reported in those with severe cases of COVID-19 [40]. Accordingly, several interleukins (e.g., IL-3, IL-6 or IL-10) have been identified as predictors of adverse events and higher mortality in COVID-19 [16,17]. The concept of interleukin inhibition has been refined in the context of COVID-19 with the IL-6 inhibitor tocilizumab being one of the first established treatment options for COVID-19. In a study of 289 hospitalized patients with COVID-19, treatment with tocilizumab has been associated with a lower likelihood of progression to the composite endpoint of mechanical ventilation or death, with a reduction in mechanical ventilation being the driving force behind the positive trial result [19].

Our study assessed the prognostic impact of IL-32 and IL-34 upon hospital admission in the context of SARS-CoV-2 infection. We found that both IL-32 and IL-34 failed to predict 28-day mortality upon univariable and multivariable hazard regression analysis alike. Our observation is in line with the findings of Bergantini et al., who found comparable levels of IL-32 in patients with different levels of COVID-19 severity in a small hypothesis-generating study. A total of 64 patients with COVID-19 were included in the study and stratified by severity of disease, based on the level of oxygenation support. Similarly, no significant differences in IL-32 and IL-34 were found with regard to PaO2/FiO2 levels upon admission in our study population [24]. According to our research findings, routine measurements of these biomarkers cannot be advocated for prediction of short-term all-cause mortality in COVID-19. Some reasons for the missing association of IL-32 and IL-34 with mortality in our study population should be discussed. First, we only measured biomarker levels upon hospital admission, which usually represents an early stage of the disease course. Depending on the underlying pathophysiological mechanisms of IL-32 and IL-34 release in COVID-19, one may suggest that these biomarkers can be of prognostic value if measured at a different time point. However, we have identified several laboratory biomarkers (most notably hs-cTnI, copeptin and MR-proANP)—also measured upon hospital admission—as predictors of survival in a similar cohort of COVID-19 patients. Biomarkers providing prognostic information at an earlier disease stage are of more value to a treating physician as they can guide initial clinical management and decision making [26,27]. Second, our study population comprised unselected patients presenting to the emergency department with subsequent hospital admission. While this represents the general hospitalized COVID-19 patient population well, it is possible that dynamics of IL-32 and IL-34 are different in pre-specified populations, such as patients requiring intensive care treatment upon admission.

Throughout the literature, CV disease has been identified as a marker of risk in COVID-19. A recent meta-analysis of pooled data showed a significantly increased risk of adverse outcomes in patients with COVID-19 and pre-existing CV disease (pooled odds ratio of 1.41 (95% CI, 1.32–1.51)) [41]. Similarly, Zhang et al. found a significantly increased risk of mortality in patients with a history of CV disease (22.2%) compared to those without CV disease (9.8%). The authors also reported an even higher mortality risk with increasing CV disease burden as patients with two or more CV diseases had a striking mortality rate of 42.5% [42]. Our findings further add to this observation, as we report a significantly higher 28-day mortality in patients with CV disease with ≥three CV diseases having the highest risk. This association persisted upon multivariable Cox regression analysis after adjusting for age and gender as well as after adjusting for age, gender and comorbidities. In addition to these findings, our study also provided input into the characteristics of patients with COVID-19 and concomitant CV disease. Evidence of hypervolemia upon chest X-ray alongside markedly elevated levels of cardiovascular biomarkers, NT-proBNP and hs-cTnI, were common findings among these patients. Our findings allude to the recommendations of a conservative fluid management strategy in the management of patients with COVID-19 and that this approach may be especially important in those with a history of CV disease at imminent risk of respiratory deterioration [43]. Interestingly, we did not find any significant differences with regard to pO2/FiO2 levels and cardiovascular disease status, which implies hypoxemia as an unlikely pathophysiological mechanism for the increased mortality with CV disease in COVID-19. As expected, we also observed more pathological features of CV disease upon admission ECG, including bundle branch block and ST-segment deviations. The typical pattern of inflammation in COVID-19, defined by neutrophilia and concomitant lymphopenia resulting in a high neutrophil-to-lymphocyte ratio, was also more pronounced among patients with CV disease. This association may be linked to the acute response to SARS-CoV-2 infection but may also allude to the commonly observed state of chronic inflammation in CV disease [44]. With regard to IL-32 and IL-34, only a modest correlation was found between neutrophils and IL-34, while CRP and lymphocytes showed no correlations with either interleukin.

This study has some limitations that should be acknowledged. Since we only included hospitalized patients with COVID-19, we cannot draw any conclusions for patients discharged or treated at home. Second, measurement of IL-32/IL-34 was done upon admission to the ED without any further measurements during the hospital stay. We can therefore not analyze the potential impact of temporal changes of these biomarkers. Since our primary endpoint was 28-day mortality, we can only make assumptions about short-term survival. The presence of comorbidities was interpreted by the treating physicians without external validation. Since only admission ECGs were included for analysis, we cannot draw conclusion towards ECG changes during the COVID-19 infection. Additionally, extended cardiac work-up (e.g., echocardiography or non-invasive/invasive coronary diagnostics) was not performed systematically, which does not allow diagnosis of CV disease from the ECG findings. We believe that our study was adequately powered to assess the primary research question of an association of IL-32/IL-34 with CV disease and mortality as the results of statistical analysis were very clear with no statistical significance or trends towards any associations. Confirmatory studies with a larger sample size and additional biomarker testing may better characterize subgroups and allow the interpretation of the timely course of IL-32/IL-34.

## 5. Conclusions

IL-32 and IL-34 were neither associated with CV disease nor with clinical endpoints in the context of COVID-19. Hospitalized patients with COVID-19 and multiple CV diseases, however, were at an increased risk of short-term mortality. Further research is needed to elucidate the pathophysiological mechanism for CV involvement in COVID-19.

## Figures and Tables

**Table 1:** Baseline characteristics and clinical outcomes of the study population stratified by CV disease. * indicates a significant difference to those without CV disease after Bonferroni correction.

Characteristics	Study Population (n = 245)	No CV Disease (n = 161)	1 or 2 CV Disease (n = 69)	≥3 CV Disease (n = 15)	*p*-Value
Baseline characteristics					
Age, years	67 (52–79)	60 (49–71)	78 (69–85) *	82 (79–91) *	<0.001
Male sex	130 (53.1%)	84 (52.2%)	35 (50.7%)	11 (73.3%)	0.262
Arterial hypertension	145 (53.1%)	75 (46.6%)	57 (82.6%) *	13 (86.7%) *	<0.001
Diabetes mellitus	74 (30.2%)	41 (25.5%)	28 (40.6%)	5 (33.3%)	0.070
Chronic pulmonary disease	33 (13.5%)	18 (11.2%)	12 (17.4%)	3 (20.0%)	0.336
Chronic kidney disease	45 (18.4%)	12 (7.5%)	26 (37.7%) *	7 (46.7%) *	<0.001
History of malignancy	37 (15.1%)	16 (9.9%)	20 (29.0%) *	1 (6.7%)	<0.001
Signs and symptoms					
Fever	183 (74.7%)	131 (81.4%)	43 (62.3%)	9 (60.0%)	0.004
Coughing	118 (48.2%)	85 (52.8%))	26 (37.7%)	7 (46.7%)	0.109
Dyspnea	141 (57.6%)	93 (57.8%)	40 (58.0%)	8 (53.3%)	0.943
Clinical Outcomes					
Hospitalization length, days	11 (8–17)	10 (7–14)	14 (10–22) *	18 (9–23)	<0.001
pO2/FiO2	286 (244–320)	287 (248–319)	283 (222–331)	279 (263–330)	0.609
28-day mortality	37 (15.1%)	11 (6.8%)	17 (24.6%) *	9 (60.0%) *	<0.001

**Table 2:** Diagnostic findings of the study population stratified by CV disease. * indicates a significant difference to those without CV disease after Bonferroni correction.

Characteristics	Study Population (n = 245)	No CV Disease (n = 161)	CV Disease (n = 69)	≥3 CV Disease (n = 15)	*p*-Value
ECG findings					
Heart rate, bpm	89 (78–105)	89 (80–102)	91 (72–101)	107 (71–120)	0.319
PQ time, ms	152 (137–170)	150 (136–162)	172 (137–192) *	167 (154–188)	0.005
QRS width, ms	97 (86–105)	96 (86–103)	97 (86–109)	101 (95–141) *	0.040
Bundle branch block	26 (11.0%)	11 (7.1%)	10 (15.2%)	5 (33.3%) *	0.003
ST-segment deviation	33 (15.5%)	14 (9.5%)	14 (25.0%) *	5 (50%) *	<0.001
QT time, ms	364 (339–397)	361 (336–385)	375 (348–410) *	366 (340–426)	0.023
Chest X-ray findings					
Infiltrate	184 (75.7%)	126 (78.8%)	47 (69.1%)	11 (73.3%)	0.293
Cardiomegaly	130 (53.5%)	76 (47.5%)	42 (61.8%)	12 (80.0%) *	0.015
Interstitial edema	38 (15.6%)	22 (13.8%)	9 (13.2%)	7 (46.7%) *	0.003
Pleural effusion	37 (15.2%)	18 (11.3%)	16 (23.5%)	3 (20.0%)	0.053
Laboratory values at admission					
White blood cells, G/L	6.9 (5.3–9.2)	6.5 (5.2–8.7)	7.5 (5.4–10.1)	8.4 (6.5–10.8)	0.055
Neutrophil granulocytes, G/L	5.2 (3.7–7.4)	4.9 (3.6–6.7)	5.5 (4.0–8.6)	6.1 (4.9–9.8) *	0.007
Lymphocytes, G/L	0.97 (0.70–1.33)	1.06 (0.8–1.5)	0.83 (0.59–1.14)	0.58 (0.53–1.01) *	<0.001
Neutrophil to lymphocyte ratio	5.1 (3.0–9.3)	4.3 (2.8–7.6)	8.1 (4.5–12.0) *	9.6 (7.3–12.6) *	<0.001
C-reactive protein, mg/L	65 (28–117)	62 (25–121)	65 (34–112)	85 (49–119)	0.631
Hemoglobin, g/dL	13.4 (12.1–14.5)	13.6 (12.4–14.6)	12.6 (11.4–14.1) *	12.9 (10.9–14.0)	0.008
Platelets, G/L	203 (166–252)	206 (163–263)	194 (168–240)	187 (163–221)	0.556
Creatinine, mg/dL	1.0 (0.8–1.3)	0.9 (0.8–1.2)	1.1 (0.8–1.6) *	1.5 (1.3–1.9) *	<0.001
Sodium, mmol/L	137 (135–139)	137 (135–138)	137 (135–140)	136 (134–140)	0.320
Potassium, mmol/L	4.0 (3.7–4.2)	4.0 (3.7–4.2)	4.0 (3.8–4.4)	4.0 (3.8–4.0)	0.238
Lactate dehydrogenase, U/L	277 (228–379)	280 (222–378)	271 (235–364)	312 (236–490)	0.434

**Table 3:** Correlation of IL-32 and IL-34 with CV and inflammatory biomarkers.

	Interleukin-34	Hs-cTnI	NT-proBNP	CRP	Neutrophils	Lymphocytes
**Interleukin-32, r**	0.332	0.045	0.114	−0.102	−0.004	−0.011
***p*-value**	<0.001	0.479	0.114	0.112	0.956	0.864
	**Interleukin-32**	**Hs-cTnI**	**NT-proBNP**	**CRP**	**Neutrophils**	**Lymphocytes**
**Interleukin-34, r**	0.332	0.015	0.041	−0.115	−0.148	0.094
***p*-value**	<0.001	0.814	0.524	0.073	0.021	0.142

**Table 4:** Association of interleukin-32, interleukin-34 and CV disease with the primary endpoint. The crude model only included the biomarker in question; Model 1 was adjusted for age and gender; Model 2 was adjusted for Model 1 and arterial hypertension, diabetes mellitus, chronic kidney disease and chronic pulmonary disease.

Statistical Model	Interleukin-32			Interleukin-34			1 or 2 CV Diseases			≥3 CV Diseases		
	HR	95% CI	*p*-Value	HR	95% CI	*p*-Value	HR	95% CI	*p*-Value	HR	95% CI	*p*-Value
Crude model	1.00	0.999–1.001	0.985	1.00	0.999–1.002	0.590	4.085	1.913–8.725	<0.001	13.173	5.425–31.985	<0.001
Model 1	1.00	1.000–1.001	0.857	1.00	0.999–1.002	0.530	1.631	0.728–3.656	0.234	4.013	1.443–11.157	0.008
Model 2	1.00	1.000–1.001	0.769	1.00	0.999–1.002	0.491	1.453	0.615–3.433	0.394	3.942	1.288–12.068	0.016

## Data Availability

The datasets generated for this study are available on request to the corresponding author.
